# Cross-Protection Against Four Serotypes of Dengue Virus in Mice Conferred by a Zika DNA Vaccine

**DOI:** 10.3389/fcimb.2019.00147

**Published:** 2019-05-08

**Authors:** Ran Wang, Na Gao, Yun Li, Dongying Fan, Zida Zhen, Kaihao Feng, Hui Chen, Jing An

**Affiliations:** ^1^Department of Microbiology and Parasitology, School of Basic Medical Sciences, Capital Medical University, Beijing, China; ^2^Center of Epilepsy, Beijing Institute for Brain Disorders, Beijing, China

**Keywords:** Zika virus, dengue virus (DENV), DNA vaccine, PrM/E proteins, cross-reactive immune responses, Zika-DNA-vaccination Confers Cross-protection to DENVs

## Abstract

Both Zika virus (ZIKV) and four serotypes of dengue virus (DENV1–4) are antigenically related mosquito-borne flaviviruses that co-circulate in overlapping geographic distributions. The considerable amino acid sequence homology and structural similarities between ZIKV and DENV1–4 may be responsible for the complicated immunological cross-reactivity observed for these viruses. Thus, a successful Zika vaccine needs to not only confer protection from ZIKV infection but must also be safe during secondary exposures with other flavivirus, especially DENVs. In this study, we used a Zika DNA vaccine candidate (pV-ZME) expressing the ZIKV premembrane and envelop proteins to immunize BALB/c mice and evaluated the potential cross-reactive immune responses to DENV1–4. We observed that three doses of the pV-ZME vaccine elicited the production of cross-reactive antibodies, cytokines and CD8^+^ T cell responses and generated cross-protection against DENV1–4. Our results demonstrate a novel approach for design and development of safe Zika and/or dengue vaccines.

## Introduction

Both Zika virus (ZIKV) and four serotypes of dengue viruses (DENV1–4) belong to the genus *Flavivirus*, family *Flaviviridae*. These viruses are antigenically related mosquito-borne pathogens that are transmitted through the same vectors, *Aedes aegypti* and *Aedes albopictus*, and co-circulate in overlapping geographic distributions. ZIKV and DENV1–4 share an overall 52–57% homology in all amino acid sequences (Barba-Spaeth et al., 2016), which contributes to their cross-reactivity in T cell and antibody responses (Wen et al., 2017; Delgado et al., 2018). Since 2016, with the spread of ZIKV in DENV-endemic regions in the north of Brazil, individuals infected by ZIKV were likely pre-exposed to DENV (Nogueira et al., 2018). This situation has aroused concern among researchers regarding the cross-reactive immune response resulting from pre-existing DENV immunity on Zika disease outcomes. Recently, several studies have indicated that pre-existing DENV immunity mediated cross-protection against secondary ZIKV infection in mice (Pantoja et al., 2017; Wen et al., 2017). Valiant et al. demonstrated that high levels of cross-neutralizing antibodies (nAbs) against DENV in ZIKV seropositive individuals could potentially reduce the enhancement of DENV infection (Valiant et al., 2018). However, some inconsistent results on the outcome of cross-reactive immune responses between DENV and ZIKV have also been reported. For example, pre-existing and highly seroprevalent anti-DENV antibodies may enhance ZIKV infection (Bardina et al., 2017; Zimmerman et al., 2018). Thus, the substantial effect of pre-existing immunity on subsequent infection by closely related flaviviruses requires further characterization in vaccinated or infected models (Priyamvada et al., 2016).

For the global fight against the ZIKV epidemic, several types of vaccine candidates have been developed, including whole inactivated, subunit, DNA and mRNA vaccines, with the immunogenicity and efficacy of these experimental vaccines having been well evaluated in mice or human (Diamond et al., 2018). However, little has been described regarding the characteristics of the cross-reactive immune responses generated by the above vaccines to subsequent infection with closely related flavivirus such as DENV, which is an important issue associated with the safety of vaccines.

We previously reported that pVAX1-ZME (pV-ZME), a Zika DNA vaccine candidate based on the premembrane and envelop proteins (prME), effectively elicited a strong protective immunity in mice (Wang et al., 2018). Furthermore, numerous similar studies have indicated that prME-based DNA vaccines can protect mice, monkeys and humans from ZIKV infection (Dowd et al., 2016; Hampton, 2016; Griffin et al., 2017). In this study, we used a murine model vaccinated with pV-ZME to investigate its cross-reactivity and protective efficacy against four DENV serotypes. Our results demonstrate that in BALB/c mice, the Zika DNA vaccine triggered cross-reactive humoral and cellular immune responses to DENV1–4 that could cross-protect mice against DENV infections.

## Materials and Methods

### Cells, Viruses, Vaccine, and Mice

Vero cells were cultured in minimum essential medium supplemented with 5% fetal bovine serum (FBS; Gibco, USA). C6/36 and DC2.4 cells were cultured in RPMI-1640 medium supplemented with 10% FBS. Rhabdomyosarcoma (RD) cells were cultured in Dulbecco's modified Eagle's medium supplemented with 10% FBS.

ZIKV (strain SMGC-1), DENV1 (strain Hawaii), DENV2 (strain Tr1751), DENV3 (strain H87) and DENV4 (strain H241) were propagated in C6/36 cells. Enterovirus 71 (EV71, strain CMU4232) was propagated in RD cells and served as an irrelevant virus control for *in vitro* experiments.

Viral particles were harvested from the culture supernatants of C6/36 or RD cells that had been infected by viruses. Subsequently, the viral particles were concentrated by 8% polyethylene glycol precipitation, purified from clarified extracts and ultracentrifuged.

The Zika DNA vaccine pV-ZME was previously manufactured by our lab, and pVAX1 (pV) served as a control (Wang et al., 2018).

Female BALB/c mice aged 6 weeks were purchased from Vital River Laboratories (China).

### Mouse Experiments

**Figure 1:**
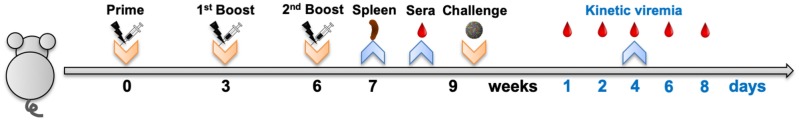
Mouse experimental workflow. Groups of mice were immunized by intramuscular electroporation with 50 μg of either pV-ZME or pV and were boosted twice at 3-week intervals. Splenocytes were obtained 1 week after the last immunization, and sera were collected 3 weeks after the last immunization. Subsequently, vaccinated mice were challenged with 1 × 10^6^ PFU of DENV1, 200 PFU of DENV2, 1 × 10^6^ PFU of DENV3, or 1 × 10^5^ PFU of DENV4. At days 1, 2, 4, 6, and 8 post challenge, the peripheral blood of mice was collected to evaluate the dynamics of viremia. Body weight changes and survival rates were observed for 21 consecutive days after challenge.

Mice were randomized into eight groups, with those in the vaccine or control groups immunized by intramuscular electroporation with either pV-ZME or pV thrice at 3-week intervals (Figure 1). Splenocytes from mice were aseptically prepared 1 week after the last immunization. Three weeks after the last immunization, sera were collected and then the mice were intracerebrally challenged with DENV1–4 individually (Figure 1). After being challenged, the peripheral blood of the mice was collected from the tail vein. During observation, mice exhibiting more than a 20% loss in weight were humanely euthanized for ethical reasons.

### Detection of Antibodies

Three weeks after the final immunization, the presence of IgG antibodies in sera were evaluated with an enzyme-linked immunosorbent assay using concentrated ZIKV, DENV1–4, or irrelevant EV71 particles as coating antigens. The highest dilution that gave an optimal density (OD) value greater than the cut-off value (half of the OD value of the negative control diluted 1:100) was recorded as the end-point titer of the IgG antibodies (Wang et al., 2018).

Similarly, the levels of nAbs were assessed using a 50% plaque reduction neutralization test (PRNT_50_) as described previously (Wang et al., 2018). The antibody titer was calculated as the geometric mean titer (GMT).

### Determination of Cytokine Profiles

The enumeration of IL-2-, IFN-γ-, IL-4-, and IL-10-producing splenocytes from mice was performed following stimulation with concentrated ZIKV, DENV1–4 or irrelevant EV71 particles using an enzyme-linked immunospot (ELISPOT) assay as previously described (Wang et al., 2018). Splenocytes (2 × 10^5^/well) were collected 1 week after the last immunization.

### Detection of IFN-γ-producing CD8^+^ T Cells

Virus-specific IFN-γ-producing cells expressing CD3e^+^ CD8^+^ IFN-γ^+^ were stained and analyzed by flow cytometry (Huang et al., 2017). Splenocytes (1.5 × 10^6^/tube) collected 1 week after the last immunization were stimulated with DC2.4 cells (0.2 × 10^5^/tube) that were pretreated with heat-inactivated ZIKV, DENV1–4, or irrelevant EV71.

### Quantification of Viral RNA

Total RNA derived from the peripheral blood after challenge was isolated using TRIzol (Sigma, USA). A primer pair targeting the *prM* region of the DENV genome (Chen et al., 2015) was used for qRT-PCR using a 7500 Real Time PCR System (Applied Biosystems, USA) and a Quant One Step qRT-PCR Kit (SYBR Green, Tiangen, China) according to the manufacturer's instructions. The proportion of mice that were viremic were calculated after challenge.

### Statistical Analysis

The data were expressed as the means ± standard deviation (SD). Statistical analyses were performed using one-way ANOVA to compare variables from different groups. The viremic reduction trends after challenge were compared using the Chi-square test. Kaplan-Meier survival curves were plotted and statistically evaluated using the Log-rank test. Probability values of ^*^P < 0.05, ^**^P < 0.01, and ^***^P < 0.001 were considered significant.

## Results

**Figure 2:**
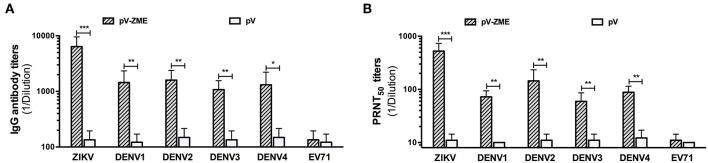
Anti-DENV antibody responses of immunized sera **(A)**. IgG antibody and **(B)** nAb titers against ZIKV, DENV1–4, and EV71. The titers of antibodies against ZIKV and EV71 served as the positive and irrelevant (non-flavivirus RNA virus) controls, respectively. Each bar represents GMTs + SD (*n* = 7). *P < 0.05, **P < 0.01, and ***P < 0.001.

First, to determine the presence of ZIKV-specific antibodies induced by pV-ZME-vaccination, an antibody assay was performed using ZIKV and EV71 as positive and irrelevant viral controls. We observed high levels of anti-ZIKV antibodies, including IgG antibodies and nAbs, in the sera of immunized mice, suggesting the induction of a ZIKV-specific humoral immune response (Figures 2A,B). Furthermore, to investigate whether the pV-ZME-vaccination elicited a cross-humoral immune response to DENV, the presence of antibodies against DENV1–4 in the sera of immunized mice was evaluated. As shown in Figure 2, compared to the corresponding controls and the irrelevant EV71 control, immunization with pV-ZME induced high and relatively uniform levels of DENV-specific cross-reactive IgG antibodies, with titers of more than 1:1,000. Moreover, sera from pV-ZME-immunized mice displayed broad cross-neutralizing potency against DENV1–4. The nAb titers toward DENV1, DENV2, DENV3, and DENV4 were 1:72.5, 1:145, 1:59.4, and 1:88.3, respectively, and were significantly different from their corresponding controls and the irrelevant control (Figure 2B, *P* < 0.01). The results demonstrated that the Zika DNA vaccine could elicit relatively balanced cross-reactive antibody responses to DENV1–4.

**Figure 3:**
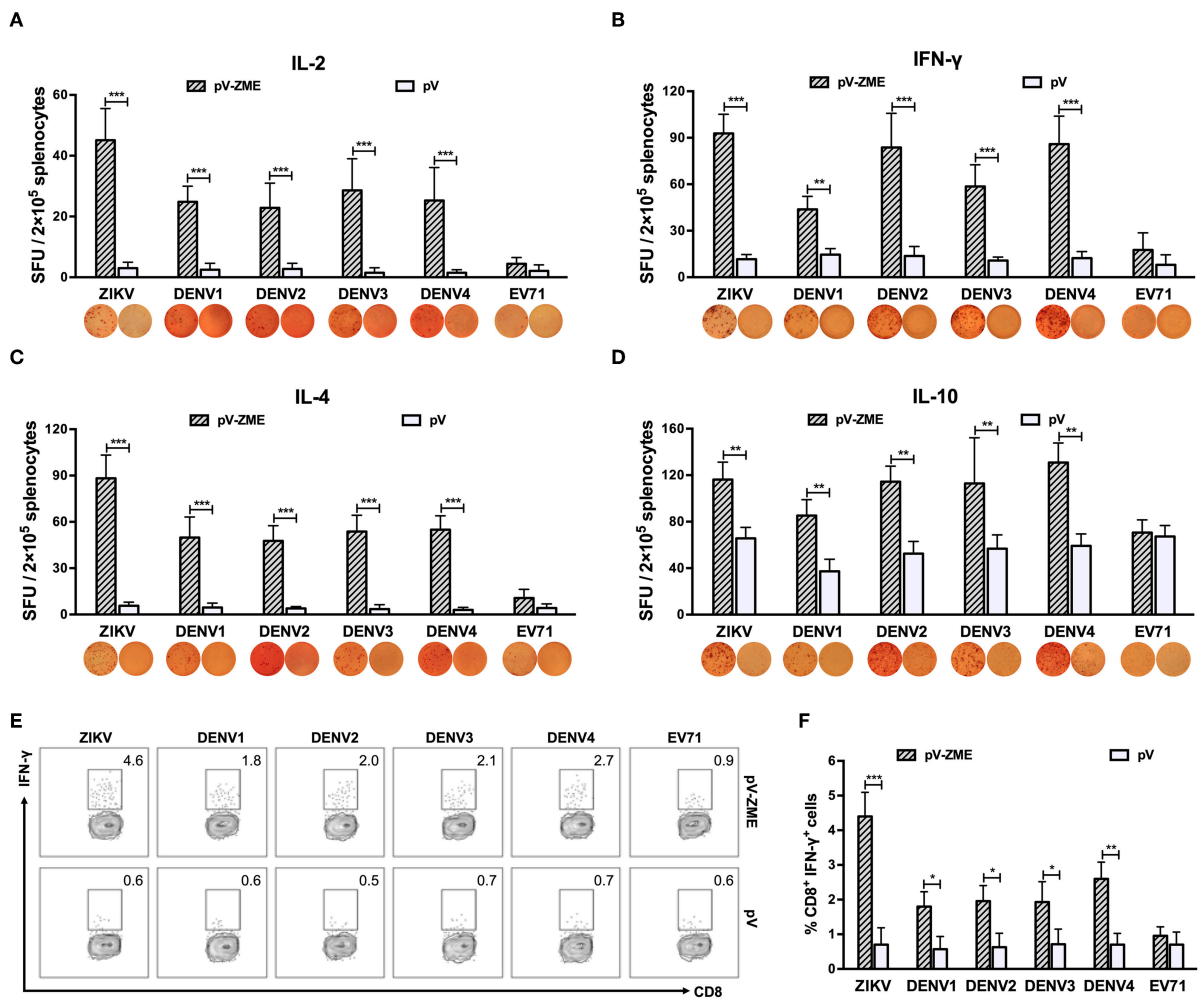
Splenocyte-secreted cytokines and frequencies of IFN-γ-producing CD8^+^ T cells in splenocytes **(A–D)**. Cytokine production by antigen-stimulated splenocytes determined by ELISPOT. One week after the last immunization, splenocytes were isolated and stimulated with ZIKV, DENV1–4 or EV71 particles. After 72 h of stimulation and culturing, the **(A)** IL-2-positive, **(B)** IFN-γ-positive, **(C)** IL-4-positive, and **(D)** IL-10-positive spots were determined. The data are expressed as the means + SD of each group with representative images. **(E, F)** Antigen-specific IFN-γ intracellular staining of CD8^+^ T cells detected by flow cytometry. **(E)** Expression of IFN-γ^+^ in CD8^+^ T cells. Splenocytes were stained with anti-CD3e FITC, anti-CD8 APC-A750, or anti-IFN-γ PE monoclonal antibodies. Dot plots show representative examples. **(F)** Percentages are expressed from the gates:CD3e^+^ CD8^+^ IFN-γ^+^ T cell areas. The results are expressed as the mean values + SD (*n* = 7). *P < 0.05, **P < 0.01, and ***P < 0.001.

Next, an ELISPOT assay was performed to examine whether pV-ZME induced secretion of Th1/Th2-type cytokines in response to ZIKV, DENV or irrelevant EV71. Upon stimulation with ZIKV particles, splenocytes from vaccinated mice produced a potent and mixed Th1 (IL-2 and IFN-γ)/Th2 (IL-4 and IL-10)-type cytokine response (Figures 3A–D, *P* < 0.01), demonstrating a positive response to ZIKV. In addition, DENV1–4 stimulation induced higher levels of IL-2, IFN-γ, IL-4, and IL-10 in the vaccine groups compared to that observed in the corresponding controls or the irrelevant control (*P* < 0.01). These results demonstrated that vaccination with pV-ZME elicited cross-reactive Th1/Th2 mixed cytokine responses to DENV1–4.

Furthermore, the production of virus-specific IFN-γ was detected in splenocytes stimulated with DC2.4 cells that had been pretreated with heat-inactivated viruses. Similar to the ELISPOT results, a large number of ZIKV-specific IFN-γ-producing CD8^+^ T cells were generated in pV-ZME-vaccinated mice (Figures 3E,F, *P* < 0.001). In addition, vaccinated mice produced relatively high frequencies of cross-reactive CD8^+^ IFN-γ^+^ cells in response to DENV antigens but not to the irrelevant control (*P* < 0.05). The results indicated that the Zika DNA vaccine elicited the production of cross-reactive antibodies and cross-reactive cell-mediated immune responses to all four DENV serotypes.

**Figure 4:**
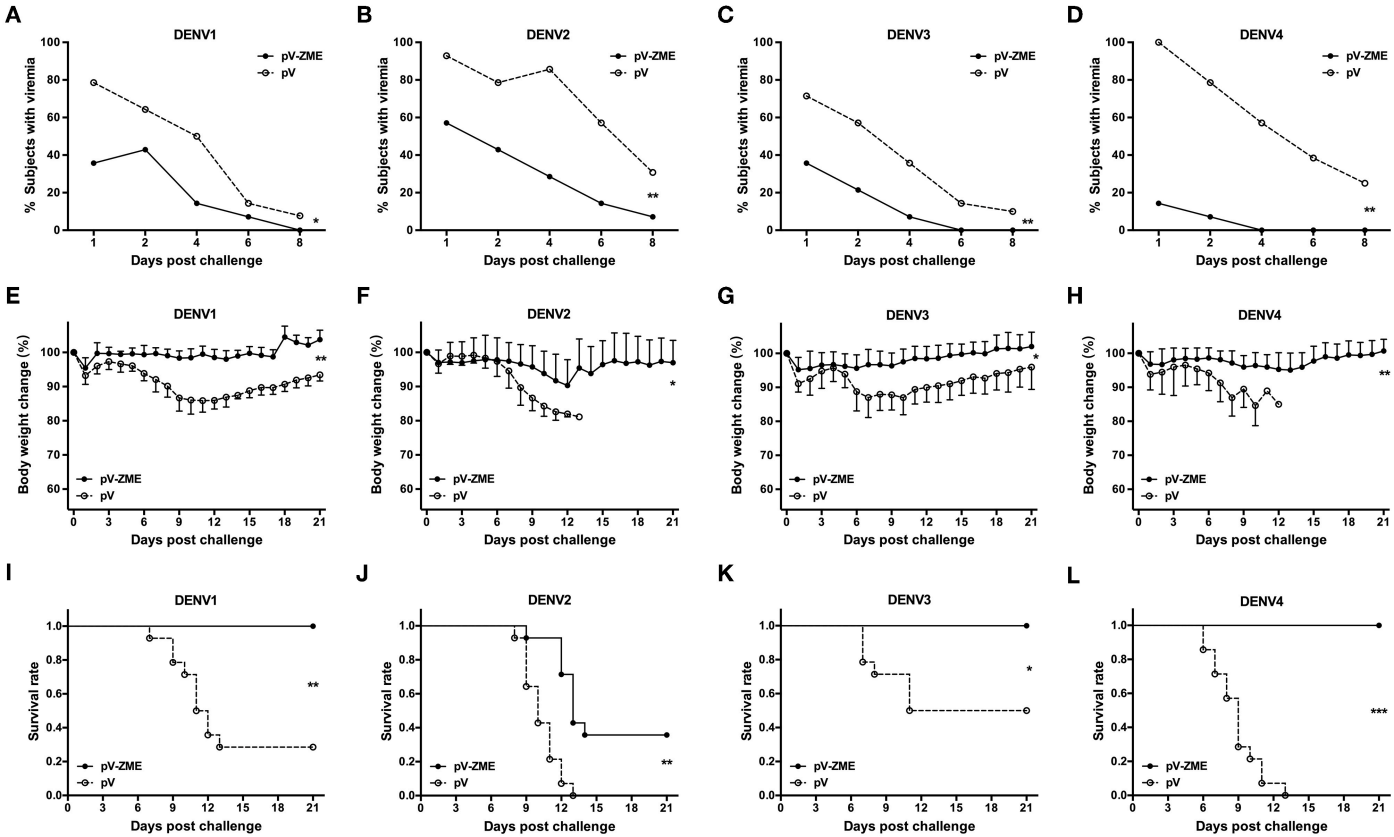
Immunization of mice with the Zika DNA vaccine pV-ZME confers a reduction in DENV viremia and cross-protection against challenge with four serotypes of DENV **(A–D)**. The kinetics of viremia was evaluated in peripheral blood on days 1, 2, 4, 6, and 8 after DENV1–4 challenge. **(E–H)** Body weight changes and **(I–L)** survival rates were monitored daily for 21 consecutive days (*n* = 14). The results are expressed as the mean values ± SD. *P < 0.05, **P < 0.01, and ***P < 0.001.

Finally, we investigated whether the observed cross-reactivity could protect immunized mice from DENV challenge. The kinetics of viremia, body weight changes and survival rates after challenge with DENVs are shown in Figure 4. In the pV group, DENV RNA was detected in 71.4–100% mice at day 1 after challenge, and viremic mice were continuously observed during the detection period (until day 8 after challenge). In contrast, mice were less viremic in the pV-ZME group, with 14.3–57.1% of mice exhibiting viremia at day 1 after challenge with DENV1–4, and the trends in viremia reduction were significantly different from those observed in the pV group (Figures 4A–D, χ^2^ = 10.15 and *P* < 0.05 for DENV1, χ^2^ = 13.35 and *P* < 0.01 for DENV2, χ^2^ = 13.99 and *P* < 0.01 for DENV3, and χ^2^ = 14.88 and *P* < 0.01 for DENV4).

Similarly, mice in the pV group showed obvious body weight losses that ranged from 13.0 to 18.9% after DENV challenge. Notably, all pV-ZME-immunized mice showed limited mean body weight loss, ranging from 4.8 to 9.7%, compared to the control mice (13.0–18.9%, Figures 4E–H, *P* < 0.01 for DENV1 and DENV4, and *P* < 0.05 for DENV2 and DENV3). Consistently, all pV-ZME-immunized mice survived being challenged with DENV1, DENV3, or DENV4 (Figures 4I–L). In contrast, survival rates of 28.6% (4/14), 50.0% (7/14), and 0% (0/14) were noted in the corresponding controls, respectively. For mice challenged with DENV2, mice immunized with pV-ZME exhibited a body weight loss of approximately 9.7% and a survival rate of 35.7% (5/14). Although the cross-protection against DENV2 in vaccinated mice was not complete, the body weight and survival rate were better than that observed for the control mice (Figure 4F, *P* < 0.05 and Figure 4J, *P* < 0.01). Taken together, our results demonstrated that pV-ZME can confer cross-protection against four DENV serotypes in mice.

## Discussion

Recently, multiple Zika vaccine candidates, especially DNA vaccines, have shown promising outcomes after undergoing successful preclinical and clinical development (Abbink et al., 2018). Because of the complicated immunological cross-reactivity among flaviviruses, it is necessary to determine the role of pre-existing ZIKV immunity in cross-reactivity to DENV infection within the same host. This information will be crucial for informing public health responses and valuable for the design and application of Zika vaccine candidates.

In this study, mice immunized with pV-ZME showed relatively high titers of cross-reactive IgG antibodies and nAbs as well as relatively high levels of Th1/Th2 mixed cytokine responses to four serotypes of DENV (Figures 2, 3A–D). Furthermore, we also showed that the frequency of cross-reactive IFN-γ-producing CD8^+^ T cells markedly increased upon stimulation with DENV antigens (Figure 3F). More importantly, transient viremia, limited body weight loss and complete survival after DENV challenge confirmed that cross-reactive immune responses induced by pV-ZME vaccination could protect mice against DENV infection (Figure 4). These results suggested that this Zika DNA vaccine candidate can induce both cross-reactive humoral and cellular immune responses to collaboratively provide cross-protection against DENV infections.

ZIKV structures have revealed its high similarity to DENV, and the two closely related flaviviruses share considerable homology in their amino acid sequences, which contains analogous surface regions associated with common epitopes. The existence of a set of common epitopes or homologous antigenic peptides is believed to be the primary reason for the cross-reactivity between ZIKV and DENV (Sirohi and Kuhn, 2017). Generally, both durable humoral immune responses and CD8^+^ T cell responses play essential roles in the efficacy of dengue vaccines (Zellweger et al., 2014; Lam et al., 2017). Recently, Wen et al. showed that the CD8^+^ T cell response induced during initial DENV infection can provide cross-protection against a subsequent ZIKV infection in mice (Wen et al., 2017). In this study, we showed that following antigenic stimulation with DENV proteins, pV-ZME was capable of generating cross-reactive IFN-γ-producing CD8^+^ T cell in response to DENV. Although non-structural (NS) proteins have been reported to contribute to inducing a strong CD8^+^ T cell response, there are also some CD8^+^ T cell epitopes within the E protein (Yauch et al., 2009) that may be responsible for inducing the cross-reactive CD8^+^ T cell response in mice vaccinated with pV-ZME. Thus, regarding future modifications of Zika vaccine design, a reasonable introduction of *NS* gene(s) may improve the cell-mediated immune response.

Furthermore, it should be noted that although relatively comparable cross-nAb titers were detected against all four DENV serotypes, a relatively limited protection against DENV2 was observed that was not significantly associated with the nAb titers in the sera of vaccinated mice, the reason for which remains unclear. Similarly, the licensed dengue vaccine CYD-TDV falls short in affording enough protection against DENV2, although it has a high neutralizing potency toward DENV2 (Capeding et al., 2014; Villar et al., 2015; Agarwal et al., 2017; Henein et al., 2017). Therefore, this phenomenon regarding DENV2 requires further attention for the development of dengue vaccines. Moreover, due to antigenic diversity between ZIKV and DENV1–4, it is reasonable that the Zika DNA vaccine confers different levels of cross-protection against DENV1–4. For a successful Zika vaccine, it is necessary to comprehensively evaluate both its protective efficacy against ZIKV infection as well as the underlying cross-reactive immune responses to closely related flaviviruses, especially DENV.

Recent studies have indicated that Zika DNA vaccine candidates expressing prM/E proteins can induce high levels of specific antibodies and protection in monkeys and mice (Dowd et al., 2016; Hampton, 2016; Griffin et al., 2017), with vaccinated animals showing reduced levels of viremia and no visible signs of illness. A DNA vaccine was previously reported to be unable to induce a significant immune response in large animal species (Dupuis et al., 2000). However, recent studies have demonstrated the effectiveness of Zika DNA vaccines in generating strong humoral and cellular immune responses in large animal models. Our DNA vaccine has shown inconsistent success in triggering relatively high levels of systemic immune responses to ZIKV and DENVs. Thus, in combination with these reports, our results suggest that pV-ZME is a promising Zika vaccine candidate for future applications. Moreover, our data provides novel and insightful information for the design and development of safe Zika and/or dengue vaccines.

In summary, the results of our study demonstrated that pV-ZME not only elicited systemic immune responses and effective protection against ZIKV (Wang et al., 2018), it conferred cross-protection to four serotypes of DENV in mice. Our results indicate the potential for the widespread use of the Zika prME-based DNA vaccine. Furthermore, it is worthwhile to identify common epitopes for the future development of a novel bivalent or multivalent recombinant subunit vaccine against both DENV and ZIKV.

## Data Availability

All datasets generated for this study are included in the manuscript.

## Ethics Statement

This study had been approved by the Institutional Animal Care and Use Committee of Chinese Capital Medical University and performed in strict accordance with Regulations for the Administration of Affairs Concerning Experimental Animals.

## Author Contributions

RW designed and performed the experiments and wrote the manuscript. NG, YL, DF, ZZ, and KF helped perform the experiments. HC analyzed the data and revised the manuscript. JA designed the experiments and revised the manuscript.

### Conflict of Interest Statement

The authors declare that the research was conducted in the absence of any commercial or financial relationships that could be construed as a potential conflict of interest.

## References

[B1] Abbink P. Stephenson K. E. Barouch D. H. (2018). Zika virus vaccines. Nat. Rev. Microbiol. 16, 594–600. 10.1038/s41579-018-0039-7 29921914 PMC6162149

[B2] Agarwal R. Wahid M. H. Yausep O. E. Angel S. H. Lokeswara A. W. (2017). The immunogenicity and safety of CYD-tetravalent dengue vaccine (CYD-TDV) in children and adolescents: a systematic review. Acta Med. Ind. 49, 24–33. 28450651

[B3] Barba-Spaeth G. Dejnirattisai W. Rouvinski A. Vaney M. C. Medits I. Sharma A.. (2016). Structural basis of potent Zika-dengue virus antibody cross-neutralization. Nature 536, 48–53. 10.1038/nature18938 27338953

[B4] Bardina S. V. Bunduc P. Tripathi S. Duehr J. Frere J. J. Brown J. A.. (2017). Enhancement of Zika virus pathogenesis by preexisting antiflavivirus immunity. Science 356, 175–180. 10.1126/science.aal4365 28360135 PMC5714274

[B5] Capeding M. R. Tran N. H. Hadinegoro S. R. Ismail H. I. Chotpitayasunondh T. Chua M. N.. (2014). Clinical efficacy and safety of a novel tetravalent dengue vaccine in healthy children in Asia: a phase 3, randomised, observer-masked, placebo-controlled trial. Lancet 384, 1358–1365. 10.1016/S0140-6736(14)61060-6 25018116

[B6] Chen H. Parimelalagan M. Lai Y. L. Lee K. S. Koay E. S. Hapuarachchi H. C.. (2015). Development and evaluation of a SYBR green-based real-time multiplex RT-PCR assay for simultaneous detection and serotyping of dengue and chikungunya viruses. J. Mol. Diagn. 17, 722–728. 10.1016/j.jmoldx.2015.06.008 26455921 PMC7106138

[B7] Delgado F. G. Torres K. I. Castellanos J. E. Romero-Sánchez C. Simon-Lorière E. Sakuntabhai A.. (2018). Improved immune responses against Zika virus after sequential dengue and zika virus infection in humans. Viruses 10:E480 10.3390/v10090480 30205518 PMC6164826

[B8] Diamond M. S. Ledgerwood J. E. Pierson T. C. (2018). Zika virus vaccine development: progress in the face of new challenges. Annu. Rev. Med. 70, 121–135 10.1146/annurev-med-040717-051127 30388054

[B9] Dowd K. A. Ko S. Y. Morabito K. M. Yang E. S. Pelc R. S. DeMaso C. R.. (2016). Rapid development of a DNA vaccine for Zika virus. Science 354, 237–240. 10.1126/science.aai9137 27708058 PMC5304212

[B10] Dupuis M. Denis-Mize K. Woo C. Goldbeck C. Selby M. J. Chen M.. (2000). Distribution of DNA vaccines determines their immunogenicity after intramuscular injection in mice. J. Immunol. 165, 2850–2858. 10.4049/jimmunol.165.5.2850 10946318

[B11] Griffin B. D. Muthumani K. Warner B. M. Majer A. Hagan M. Audet J.. (2017). DNA vaccination protects mice against Zika virus-induced damage to the testes. Nat. Commun. 8:15743. 10.1038/ncomms15743 28589934 PMC5467228

[B12] Hampton T. (2016). DNA vaccine protects monkeys against zika virus infection. J. Am. Med. Assoc. 316:1755. 10.1001/jama.2016.15862 27802535

[B13] Henein S. Swanstrom J. Byers A. M. Moser J. M. Shaik S. F. Bonaparte M.. (2017). Dissecting antibodies induced by a chimeric yellow fever-dengue, live-attenuated, tetravalent dengue vaccine (CYD-TDV) in naive and dengue-exposed individuals. J. Infect. Dis. 215, 351–358. 10.1093/infdis/jiw576 27932620 PMC6392503

[B14] Huang H. Li S. Zhang Y. Han X. Jia B. Liu H.. (2017). CD8(+) T cell immune response in immunocompetent mice during Zika virus infection. J. Virol. 91:e00900–17. 10.1128/JVI.00900-17 28835502 PMC5660488

[B15] Lam J. H. Chua Y. L. Lee P. X. Martínez Gómez J. M. Ooi E. E. Alonso S. (2017). Dengue vaccine-induced CD8+ T cell immunity confers protection in the context of enhancing, interfering maternal antibodies. JCI Insight. 2:94500. 10.1172/jci.insight.94500 29263304 PMC5752305

[B16] Nogueira M. L. Nery Júnior N. R. R. Estofolete C. F. Bernardes Terzian A. C. Guimarães G. F. Zini N. Alves da Silva R.. (2018). Adverse birth outcomes associated with Zika virus exposure during pregnancy in Sao Jose do Rio Preto, Brazil. Clin. Microbiol. Infect. 24, 646–652. 10.1016/j.cmi.2017.11.004 29133154

[B17] Pantoja P. Pérez-Guzmán E. X. Rodríguez I. V. White L. J. González O. Serrano C.. (2017). Zika virus pathogenesis in rhesus macaques is unaffected by pre-existing immunity to dengue virus. Nat. Commun. 8:15674. 10.1038/ncomms15674 28643775 PMC5490051

[B18] Priyamvada L. Quicke K. M. Hudson W. H. Onlamoon N. Sewatanon J. Edupuganti S.. (2016). Human antibody responses after dengue virus infection are highly cross-reactive to Zika virus. Proc. Natl. Acad. Sci. U.S.A. 113, 7852–7857. 10.1073/pnas.1607931113 27354515 PMC4948328

[B19] Sirohi D. Kuhn R. J. (2017). Zika virus structure, maturation, and receptors. J. Infect. Dis. 216, S935–S944. 10.1093/infdis/jix515 29267925 PMC5853281

[B20] Valiant W. G. Lalani T. Yun H. C. Kunz A. Burgess T. H. Mattapallil J. J. (2018). Human serum with high neutralizing antibody titers against both zika and dengue virus shows delayed in vitro antibody-dependent enhancement of dengue virus infection. Open Forum Infect. Dis. 5:ofy151. 10.1093/ofid/ofy151 30019003 PMC6041987

[B21] Villar L. Dayan G. H. Arredondo-García J. L. Rivera D. M. Cunha R. Deseda C.. (2015). Efficacy of a tetravalent dengue vaccine in children in Latin America. N. Engl. J. Med. 372, 113–123. 10.1056/NEJMoa1411037 25365753

[B22] Wang R. Liao X. Fan D. Wang L. Song J. Feng K.. (2018). Maternal immunization with a DNA vaccine candidate elicits specific passive protection against post-natal Zika virus infection in immunocompetent BALB/c mice. Vaccine 36, 3522–3532. 10.1016/j.vaccine.2018.04.051 29753607

[B23] Wen J. Elong Ngono A. Regla-Nava J. A. Kim K. Gorman M. J. Diamond M. S.. (2017). Dengue virus-reactive CD8(+) T cells mediate cross-protection against subsequent Zika virus challenge. Nat. Commun. 8:1459. 10.1038/s41467-017-01669-z 29129917 PMC5682281

[B24] Yauch L. E. Zellweger R. M. Kotturi M. F. Qutubuddin A. Sidney J. Peters B.. (2009). A protective role for dengue virus-specific CD8+ T cells. J. Immunol. 182, 4865–4873. 10.4049/jimmunol.0801974 19342665 PMC2674070

[B25] Zellweger R. M. Eddy W. E. Tang W. W. Miller R. Shresta S. (2014). CD8+ T cells prevent antigen-induced antibody-dependent enhancement of dengue disease in mice. J. Immunol. 193, 4117–4124. 10.4049/jimmunol.1401597 25217165 PMC4185219

[B26] Zimmerman M. G. Quicke K. M. O'Neal J. T. Arora N. Machiah D. Priyamvada L. Kauffman R. C. Register E. Adekunle O. Swieboda D.. (2018). Cross-Reactive Dengue Virus antibodies augment Zika virus infection of human placental macrophages. Cell Host Microbe. 24:e736. 10.1016/j.chom.2018.10.008 30439342 PMC6394860

